# From office to digital primary care services: analysing income-related inequalities in utilization

**DOI:** 10.1186/s12939-024-02184-6

**Published:** 2024-04-30

**Authors:** Jens Wilkens, Hans Thulesius, Björn Ekman

**Affiliations:** Department of Clinical Sciences, Lund University, Malmö, Sweden Jan Waldenströms gata 35, 20205

**Keywords:** Primary care, Digital health services, Service utilization, Equality, Concentration index, Register data, Sweden

## Abstract

The use of digital technologies to deliver primary health care has increased over the past decade. While some technologies have been shown to be medically effective and efficient, the effects of digital primary care on the policy goal of equality in the use of such types of care have not been studied using large register data. The aim of this study was to analyse how digital contacts differ from officebased visits by income as an indicator of socioeconomic status. Specifically, we estimated differences in primary care utilization across income, factors of contribution to these inequalities, and applied a needs-based standardisation of utilization to estimate differences in equity.

We used a purposively built consultation level dataset with 726 000 Swedish adult patients diagnosed with an infection, including clinical and sociodemographic variables. Applying concentration indexes (CI) and graphical illustrations we measured how the two types of services are distributed relative to income. We estimated how much of the inequalities were attributed to different sociodemographic factors by decomposing the concentration indexes. Standardised utilization for sex, age and comorbidity allowed for the estimation of horizontal inequity indexes for both types of services.

Utilization by the two types of care showed large income inequalities. Office-based visits were propoor (CI -0.116), meaning lowincome patients utilized relatively more of these services, while digital contacts were prorich (CI 0.205). However, within the patient group who had at least one digital contact, the utilization was also propoor (CI -0,101), although these patients had higher incomes on average. The standardised utilization showed a smaller prorich digital utilization (CI 0.143), although large differences remained. Decomposing the concentration indexes showed that education level and being born in Sweden were strong attributes of prorich digital service utilization.

The prorich utilization effects of digital primary care may risk undermining the policy goals of access and utilization to services regardless of socioeconomic status. As digital health technologies continue to expand, policy makers need to be aware of the risk.

## Introduction

The emerging digital health technologies to deliver services raises questions about its effects on several key performance dimensions, and emerging evidence suggests that these services can provide quality and cost-effectiveness opportunities. Distributional effects across the population, how utilization relates to demography, socio-economic status and health care needs also belong to the key concerns, which need to be understood for future development and regulation. There are many reasons why patients’ opportunity to interact with primary care providers through digital tools may lead to a different utilization pattern than for traditional services. For example, as age is decisive for health care needs, and correlates with digital literacy, the transformed means to access health care providers created by digital channels are likely to impact utilization.

There is a small but growing evidence base on the effects of digital primary care on the use of services across demographic and socio-economic groups. Clearly, younger people use more digital services than others [1, 2], which has also been shown in Sweden’s neighbouring country Finland [3, 4]. For other factors, global evidence is more inconclusive. There are examples of high income being associated with higher utilization [5], but others have not been able to establish any such link [1, 6]. Global evidence is also inconclusive with regards to whether inhabitants of rural or urban areas use more digital health services. Patients with rural residence, or long distance to a provider, do use relatively more digital services according to some studies [1, 6, 7], while others show the opposite or inconclusive results [5, 8, 9]. More recently, Doty et al. point out that more evaluations of effects across socio-economic levels are needed following several countries’ attempts to incentivize digital primary care [10].

In Sweden, several studies have documented that early on when digital primary care options became available, utilization among wealthy, urban, female, and young population groups was unproportionally high [11], [12]. That there was an actual difference in utilization as compared to traditional office-based consultations was also shown in several studies, with more visits among young, higher income, urban, and people born in Sweden [13–15]. These observations have been confirmed in a study that estimated the relative importance of socio-economic factors for utilizing digital primary care [16].

When differences in care needs are considered, the comparison is further complicated. Interest in digital care applications has been shown to be lower among multimorbidity patients [17] and in patients with low education [18]. We found no studies that have explicitly applied an equity, or needs-based methodology, to evaluating utilization of digital primary care services beyond standardizing income and education groups by age and sex before comparing average utilization.

A corner stone in Sweden’s and several other countries’ health legislation is that health utilization shall be based on need, which implies that ability-to-pay should not be decisive in who uses health services [19]. To improve the understanding of the distribution of digital primary care utilization, the aim of this study was to analyse how digital contacts differed from traditional office-based visits by income and other socio-economic factors in the early phase of digital primary care.

The specific objectives were to:

1. Estimate equality in service utilization across income for the two types of primary care contacts;
2. Estimate factors of contribution to these inequalities in utilization;
3. Estimate horizontal equity in utilization for the two types of primary care contacts by applying a needs-based standardisation of utilization.

### What defines equality and equity?

The body of literature on equality and equity of health care utilization is large and clearly the two terms can be defined in various ways. The distribution of health care utilisation is of interest for several reasons. Utilisation can be more medically effective when consumed by those in most need, or those who can gain most health from the utilization. The resources used to provide the service are also used more cost-effectively if those who can gain the most consume them. But ultimately, in the context of this study, the key interest is the strive for fairness, through a needs-based utilization of health care resources, a central objective in many countries.

A common and well recognised interpretation of equality in health care is to measure how utilization relates to income, as an indicator of individuals’ ability to pay for health care services. Equal utilization is then present when health care consumption is equal across income levels [20]. The most commonly used alternative is probably education, which can be argued is more relevant for studies where many of the subjects are above working age [21].

Equity in utilization is more difficult and requires incorporating and operationalising a normative value judgement about what is a fair distribution [22]. Further, this phrase does not have an obvious practical definition, even though the concept of fairness in resource use has been applied by for example WHO [23]. If there had been a perfect measurement of care need, utilization distributed evenly across this need could be defined equitable [24]. However, it can also be argued that equity is achieved when utilization is distributed across an equal ability to benefit from that care, not the care need itself. Or even that the equal distribution of interest is the final attainment of health status [25], which can be seen as a higher ambition. Yet other aspects that can be considered are differences in preferences about health status or procedures available to meet this status [26]. Ultimately, the various definitions are dependent on a value judgement and thereby represent a variety in desired objectives [27].

Horizontal equity, that individuals with equal need are provided equal treatment irrespective of socio-economic characteristics or ability to pay, has become the most common approach in empirical literature on utilization, applied in multiple studies on equity in primary care [28], [29]. The precise measures will differ in their applications depending on the definition of need and availability of data. For example, health status as a need variable is in some studies survey-based and self-assessed and in others based on diagnoses from medical records. In this study, we applied the horizontal equity approach by adjusting all individuals’ utilization across the population by the need variables sex, age, and a diagnosed-based co-morbidity index.

## Materials and methods

### Data

We used a Swedish consultation level dataset for the calendar year 2018, collected for a project on the effects of the use of digital primary care in the Swedish health system [30]. The dataset combines clinical data on traditional office-based and digital contacts by residents in both urban, sub-urban and rural areas in seven Swedish regions purposefully chosen to represent differences across the country. These regions had a total population of 3.2 million, almost a third of Sweden’s 10.2 million population. Because utilization relative to income is the primary factor of study, and child care has different access points and co-payment rules in the regions, only patients from age 18 and older were included in the analysis, a total of 726 087 patients.

**Fig. 1:**
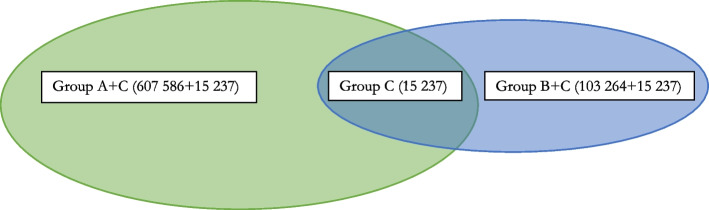
Venn diagram describing the volume of contacts by type in the data sample ^**^Groups **A**, **B** and **C** refer to Table 1

The sample included all patients who had been diagnosed with at least one of three different types of infections during the study period: urinary tract infections, upper respiratory tract infections, and skin and soft tissue infections. This diagnosis-based selection was made early in the project, because mild infections were the main reasons for contacting digital providers when this service was made available [11]. The diagnosis-based sample also means non-users are not in the material. A national identification number enabled linking of data from different sources on the level of each individual. Data on the socio-demographic variables sex, age, income, education, residency, employment status and country of birth of these individuals were collected from Statistics Sweden and follow their standard official definitions and categorization [31]. Income was defined as the individual’s gross income for the calendar year 2018, hence was neither related to other household income, nor equivalised for household composition. Data on diagnoses from specialised care (outpatient and inpatient) were collected from the National Board of Health and Welfare and used to construct a Charlson comorbidity index (CCI) value for each patient. The index values were then grouped in values zero (full health), one diagnosis, and multimorbidity (including severe or multiple diagnoses). The CCI was originally developed to classify comorbidity for estimating the risk of death [32], but has since been extensively used and validated for predicting utilization and cost [33] and gives different weights to conditions in order to reflect this. We used the most recently suggested diagnosis-list adopted to the Swedish context [34]. The final sample is described in Table 1 and the Venn diagram in Fig. 1.

**Table 1:** Descriptive statistics of sample

		**Office-based contacts only (Group A)**		**Digital contacts only (Group B)**		**Both types of contact (Group C)**	
Variable		Number	Percentage or SD*	Number	Percentage or SD*	Number	Percentage or SD*
Number of patients		607 586	(83.7%)	103 264	(14.2%)	15 237	(2.1%)
Average number of contacts		4.4	(6.3)	1.5	(1.3)	6.3	(7.0)
Annual gross income, mean SEK		212 003	(266 929)	294 485	(920 678)	225 518	(200 408)
Age, years, mean (SD)		54.0	(20.0)	35.9	(12.6)	35.3	(13.8)
Sex							
Women		341 116	(56.1%)	67 494	(65.4%)	11 067	(72.6%)
Men		266 47	(43.9%)	35 77	(34.6%)	4 17	(27.4%)
Charlson co-morbidity index groups							
Full health	517 578		(85.2%)	95 036	(92.0%)	14 058	(92.3%)
One diagnosis	42 271		(7.0%)	3 838	(3.7%)	738	(4.8%)
Multimorbidity^a^	12 008		(2.0%)	468	(0.5%)	100	(0.7%)
Missing	35 729		(5.9%)	3 922	(3.8%)	341	(2.2%)
Education level							
Elementary	138 711		(22.8%)	8 934	(8.7%)	1 702	(11.2%)
High school	275 738		(45.4%)	39 665	(38.4%)	7 006	(46.0%)
University	184 232		(30.3%)	53 97	(52.3%)	6 473	(42.5%)
Missing	8 905		(1.5%)	695	(0.7%)	56	(0.4%)
Country of birth							
Foreign	111 077		(18.3%)	15 132	(14.7%)	1 675	(11.0%)
Sweden	496 509		(81.7%)	88 132	(85.3%)	13 562	(89.0%)
Geographic region							
Rural	141 804		(23.3%)	4 976	(4.8%)	2 467	(16.2%)
Sub-urban	202 07		(33.3%)	28 547	(27.6%)	4 068	(26.7%)
Urban	263 712		(43.4%)	69 741	(67.5%)	8 702	(57.1%)
Employment status							
Non-employed	276 990		(45.6%)	14 720	(14.3%)	2 955	(19.4%)
Employed	330 596		(54.4%)	88 544	(85.7%)	12 282	(80.6%)

^*^ SD, Standard Deviation. Pearson's Chi2-test for the categorical variables and ANOVA for the continuous variables show statistical differences between the three groups (*p*-value < 0.001) for all variables

^a^ Individuals with more than one diagnosis recorded in the national patient register

Source: Digital primary care study; consultation level data for sampling period

During 2018, 607 586 individuals in the sample regions made at least one office-based visit and no digital contact (83.7% of sample, group A in Table 1 and Fig. 1), and had on average 4.4 visits. Another 103 264 people (14.2%, group B) made only digital visits and had on average 1.5 contacts. A smaller group of 15 237 (2.1%) people utilized both types of services (group C). This group had on average 6.3 contacts, of which 4.3 were office-based visits and 2.0 were digital contacts (not shown). In total, 726 087 people had at least one contact with primary care, which is 23% of the population in the seven regions. People in group B (digital contacts only) were considerably younger and had substantially higher income compared with those in group A. Utilization was generally higher among women. The difference was even larger for digital contacts than office-based primary care, mainly due to the infection diagnoses sample with a large group of women with urinary tract infections. The data also show a lower share of patients with co-morbidity, a foreign birth place, residence in rural areas and low education among the digital primary care users.

### Methods

The first specific objective, to measure equality in service utilization across income for the two types of primary care contacts, was met by applying a concentration index and a two-dimensional graphical illustration with concentration curves. The concentration index (Eq. 1) measures the accumulated utilization of the service across the population ranked from lowest to highest income, defined as two times the covariance of the number of visits (u) and the relative fractional rank of the i^th^ individual in the income distribution (R), divided by the mean of u (μ) [20].

1 $$\mathrm{Concentration\, Index }= {2}_{cov}\,({{\text{u}}}_{{\text{i}}}, {{\text{R}}}_{{\text{i}}})/\upmu .$$

**Fig. 2:**
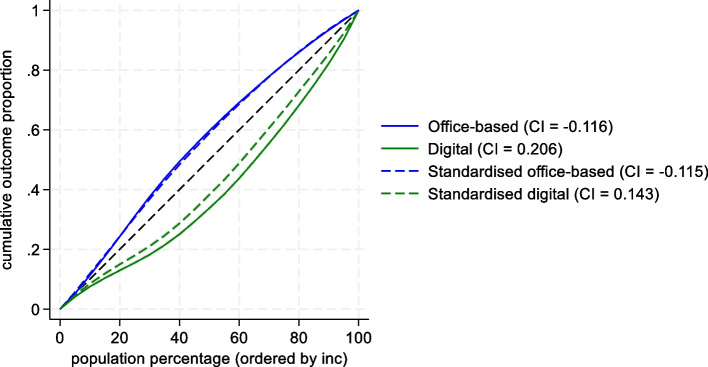
Concentration curves and concentration indexes (CI) for crude and needs-standardized office-based and digital primary care utilization across income (*n* = 726 087)

This results in a value 0 if individuals regardless of income have the same utilization, which in a graphical illustration is represented by the 45-degree line (see Fig. 2 in Results section below). A concentration index below 0 means utilization was higher among low-income individuals and graphs a curve above the diagonal line, a value above 0 describes the opposite situation with pro-rich utilisation of the studied service.

**Fig. 3:**
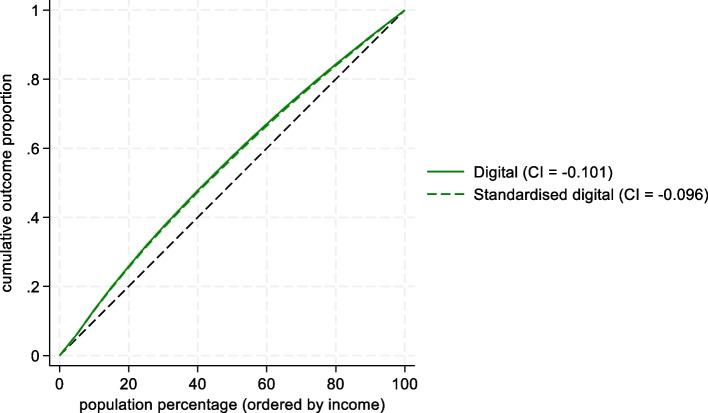
Concentration curve and index of utilization across income for patients with minimum 1 digital contact (*n* = 118 501)

Concentration indexes were first measured for both types of consultations across the entire sample, i.e., all patients were included in the same income ranking and their utilization by type of service was separately accumulated. This illustrated differences between the two services across income. Next, the same index was applied for two separate groups of patients to measure the distribution within groups. One estimate for patients who had used office-based services at least once (groups A + C in Table 1) and another who had used digital services at least once (groups B + C, see results in Fig. 3). Hence, there was an overlap of patients who had used both types of consultations (group C).

To meet the second specific objective, we estimated factors of contribution to the income inequalities in utilization measured per above. The index was decomposed into how much contribution to the income inequality can be attributed to a set of factors (defined by the variables presented in Table 1), by applying a decomposing (Eq. 2) applicable for any linear regression model with a dependent health outcome or utilization variable (*y*) and independent variables (*x*) with estimated regression coefficients (*β*) [35]. The total concentration of utilization, $C$, with respect to income, is the sum of each factors’ concentration index, ${{\Sigma }_{k}C}_{k}$. Further, the weight of each factors’ specific concentration index is given by the estimated beta coefficient $\beta$ in the ordinary least square regression, where $\overline{x }$ is the mean of the respective factor and $\mu$ is the mean of contacts. The last term is a residual component, reflecting the income-related inequality in utilization that is not explained by systematic variation in the available factors. In a perfectly specified model this estimate would be zero.

2 $$\mathrm C={\mathrm\Sigma}_{\mathrm k}{(\mathrm\beta}_{\mathrm k}\overline{\mathrm x}/\mathrm\mu){\mathrm C}_{\mathrm k}+{\mathrm C}_{\mathrm\varepsilon}/\mathrm\mu$$

This decomposing technique proposed by [35] has been widely used and is transparent in its relatively simple computation and interpretation. We note that it has been criticised for carrying a set of assumptions and that the literature presents many alternative approaches to decomposition. The concentration index is a bivariate rank dependent index, i.e. it relates individuals’ level of utilization to their relative income rank (as applied in this study). Indeed, the applied decomposition method assumes *rank ignorability*, i.e., it decomposes the utilization part of the covariance and ignores the association between the covariates and rank [36]. This means that for any explanation of changes in covariates, the income rank is assumed to remain the same, which may be troublesome as factors that impact utilization often also impact income [37]. The method also assumes exogeneity, i.e. that the error term is uncorrelated with the independent variables [38]. Hence, our chosen method is descriptive and cannot claim causality even though most literature where its applied uses language like a *factor is contributing* to a certain part of inequality. However, the several alternatives proposed are, although more sophisticated computationally, also more difficult in their interpretations, as acknowledged by their authors [36, 38].

For the third specific objective, to estimate horizontal equity, we applied indirect standardised utilization for need before relating it to income in a concentration index, which can be either pro-poor or pro-rich the same way as crude inequalities. The approach is referred to as the horizontal inequity index, which takes the value zero when individuals with the same need utilize the same volume of care irrespective of their income [39].

An ordinary least square regression estimates the parameters α, β, and γ (Eq. 3). Utilization was standardised for age, sex and co-morbidity by the need-variables $({x}_{j})$. Further, to avoid biased estimates of the need variables, the non-need variables $({z}_{k})$ country of birth, education level, employment status and geographic region were used as controlling factors. For example, not controlling for education level could have led to that the model estimated a higher needs adjustment through the correlation with our health status variable, co-morbidity. While education is correlated with utilization, we only want standardization of what we have defined as need. The remaining variation should come through the variable income in the estimation of inequity by the concentration index of needs-adjusted utilization [29].

3 $${y}_{i}= \alpha +{\sum }_{j}{\beta }_{j} {x}_{ji}+ {\sum }_{k}{\gamma }_{k} {z}_{ki}+ \varepsilon$$

Then the estimated parameters, the individual values of the standardizing variables, and sample means of the controlling variables are used to predict the utilization of each individual $({\widehat{y}}_{i})$ in the sample. Finally, the standardised utilization $({\widehat{y}}_{i}^{is})$ is equal to the individual’s utilization minus the predicted, plus the population average (Eq. 4). This indirect standardisation is the model’s interpretation of needs-based utilization.

4 $${\widehat{y}}_{i}^{is}={y}_{i}- {\widehat{y}}_{i}+ \overline{y }$$

Unlike most studies comparing equity for different types of care, commonly to answer if primary care is more or less equitable than hospital services, our two types of contacts are (arguably) intended to meet the same need. Therefore, for the comparison of utilization across the entire sample, we sought to standardise the utilization of both services by the same scale of need and calculated the needs-standardised utilization by total contacts for every patient. Standardising utilisation of the two services separately would have risked that young, well-educated and healthy individuals would have seemed to need relatively more (digital) services than others simply because this is how the service was consumed. After standardization the concentration indexes were calculated separately for all patients who had at least one office-based visit in one group (groups A and C in Table 1), and all patients who had at least one digital visit in another (groups B and C in Table 1). This way, the needs standardisation of each patient’s utilization was the same regardless of what type of visits the patient had, and the income rank was the same, which made the distributions comparable across the same income rank as for the two first crude concentration indexes by Eq. (1). For the separate analysis of utilization and income distribution comparing the groups using only one or the other type of service (results in Fig. 3), we standardised utilization solely by individuals in the respective groups.

## Results

Comparing all patients’ utilization by the two types of care showed large income inequalities. Low-income patients were more frequent users of office-based visits while high-income patients used more digital contacts (see Fig. 2). The digital use was even further from equal use on the pro-rich side (Concentration index 0.205^1^) than the office-based visits on the pro-poor side (Concentration index -0.116).

The standardised utilization to measure equity showed that when adjustment for need is applied, utilization among those who used digital contacts were considerably less pro-rich (Concentration index 0.143), while the needs-adjustment does not change the distribution of office-based utilization substantially.

However, within the respective two groups of office-based and digital service users, utilization was pro-poor in both types of care. For those 622 823 patients who had at least one office-based visit (groups A and C in Fig. 1), the concentration index was -0.073 (not shown). Standardizing utilization within this group did only marginally change the distribution (concentration index -0.068).

Among the 118 501 patients who had at least one digital contact (groups B and C in Table 1), the concentration index value was -0,101 (see Fig. 3), i.e., utilization was distributed even more pro-poor across income within the group of digital users than within the office-based users. Standardizing utilization within the digital users group also had little effect (concentration index -0.096). The crude concentration index value for the 103 264 ‘only-digital’ patients was -0.018 (not shown). These patients have considerably higher income on average, but within the group, utilization is also pro-poor, although close to equally distributed.

We also ran all standardizations without the non-need z-variables (Eq. 3) to see if only including the need variables age, sex and co-morbidity changed the results. As expected, the standardization effect, i.e. the difference to the crude inequality indexes, were then larger but the difference was very small (not shown).

Decomposing the unequal utilization by types of primary care presented in Fig. 2 explained some of the inequality. For office-based visits the model specification could explain just above half of the pro-poor inequality in utilization (0.061 of the 0.116 index value, Table 2), of which employment status contributes to half due to large income inequality and sensitivity to utilization. On the contrary, due to a negligible income effect, differences in age did not contribute to inequality, even though it was strongly associated with utilization of office-based visits.

**Table 2:** Decomposition of concentration indexes (CI) by type of primary care

Factor^a^	Office-based visits			Digital contacts		
	**Contribution**	**Weight**	**Factor specific CI**	**Contribution**	**Weight**	**Factor specific CI**
**Age**	-0.006	0.581	-0.010	0.019	-1.882	-0.010
**Sex**	-0.003	-0.048	0.055	-0.007	-0.124	0.055
**CCI**	-0.003	0.038	-0.072	0.000	0.001	-0.072
**Education**	-0.013	-0.228	0.056	0.023	0.401	0.056
**Country**	-0.003	-0.042	0.060	0.022	0.366	0.060
**Geography**	-0.003	-0.193	0.014	0.013	0.914	0.014
**Employment**	-0.032	-0.160	0.200	0.013	0.067	0.200
**Sum**	-0.061			0.083		
**Total CI**	-0.116			0.205		
**Residual**	-0.055			0.122		

^a^For full names and categories used, see Table 1

The large pro-rich inequality among digital contacts is explained to a smaller degree (0.083 of 0.205, Table 2). High education level and being born in Sweden were the factors relatively strongly associated with the pro-rich inequality in digital contacts. For both factors, income inequality was high and there was a large sensitivity to utilization.

## Discussion

In this study we find that utilization of new digital primary care services is unequally distributed in the population with a clear pro-rich pattern, while our results show a clear pro-poor distribution of traditional office-based services, the latter in line with earlier studies. But the results also reveal that within the group of digital service users, the distribution is pro-poor. One interpretation is that primary care provided by digital means, once patients have started using it, has potential to be distributed in a similarly pro-poor way as traditional primary care.

There is a contradiction in that rural and elderly patients, who ought to have the most to gain from digital services due to its travel-free nature, were using them less than others. There should be large gains in bridging this gap, although these factors had little contribution to the unequal utilization across income. For elderly, the lower use is intuitive as digital literacy is generally lower among this group [40]. Qualitative studies have found elderly in Sweden are ambivalent to using digital services and that they are also more hesitant to the privately driven digitalisation of primary care [41]. Continuity in personal contacts are also valued higher among elderly [42], which has been more difficult with digital-only service providers.

The inconclusive results in global literature indicates that the specific context and type of services are decisive factors for how utilization varies across population groups. A case in point is the Danish national digital patient portal (sundhed.dk) designed as an entry point to meet a wide range of health purposes in the population. An evaluation found no difference in overall use of the portal by sex, age, education and self-rated health. But the authors conclude that there might still be large inequalities in sub-groups of different conditions, as well as inequities, if utilization had been measured in relation to specific needs [43].

All studies above in practice used a normative approach by comparing digital services with traditional office-based care. But even though the latter is the starting point available for comparison, it cannot be assumed that traditional primary care is distributed optimally across the population. Also, not all differences between population groups in digital primary care necessarily mean utilization is less equal than in the office-based alternative. The digital contacts may complement the traditional visits by various aspects, e.g. by condition, in a way that makes them more equal. If young people consume a lot more digital care within a specific diagnosis, it may be that they have few contacts in traditional primary care for the same diagnose. There may even be a link, so that these digital contacts were made because there was underutilization of some kind in the previously only available alternative.

The observed inequalities in utilization of digital primary care can also be seen in the light of a long-lasting debate in Sweden about a more demand driven care. Health care with low or inadequate regulation has always been prone to inequities, perhaps most famously described by “the inverse care law” [44]. The differences to the pre-existing alternative office-based visits across population characteristics indicate that the relation between need and use is not the same for the two types of services. As a result of increased patient choice, especially after the 2010 legislation that liberalised the rules for where and how new primary care facilities could be established, a shift towards less pro-poor utilization was observed [45–47]. It has been frequently suggested that the digital primary care market have further exacerbated this [48]. Importantly however, these arguments build on the digital care market development, not digital service provision per se. It is difficult to empirically separate what is caused by market conditions and what is due to a newly adopted technology, especially as the development was driven by new private digital-only providers in the early phase of digital primary care in Sweden, well into our study’s time-period.

Similarly, we were not able to separate possible supply-side effects in this analysis. One of these is the risk that a supply-induced demand exists, for example by digital-only providers marketing their services more heavily in urban areas with a more affluent population.

The study is undertaken on data from before the COVID pandemic. Related to distributional effects in utilization, the question is then how socio-economic aspects interacted with the increase in digital primary care during the pandemic. The long-term effects are difficult to assess and probably local context matters a lot. For example, one study concludes that even though older and low-income patients seemed to have increased remote utilization during the pandemic, different groups did so by different means (phone, web, chat) and these modalities have varying implications for the relevance and quality of care [49].

Further on limitations, we recognise that the sampling defined by users of primary care, i.e., not including non-users, may have implications for our interpretation of utilization relative to income. We did not capture the distribution of the probability of a visit, as all observations have at least one visit of some type. Instead, we measured the visit frequency of both types of contact, conditional on at least one contact of any kind. It has been shown earlier that the conditional number of visits (non-zero) favours the poor in most OECD countries. The probability of seeing a doctor (i.e. making at least one visit) is distributed equally or pro-rich across income. But lower income patients, once they do see a GP, are likely to consult more often [39, 50, 51]. While the latter is confirmed for office-based visits in our study, we do not know if the former is true also for our context, and if so, what it looks like for digital services.

We note that our sample is a set of common infectious diseases, as these were the absolutely dominating conditions for digital services. We cannot extend the interpretation of our results beyond these conditions, or how it varies between them. Further research on chronic conditions should therefore follow.

We used crude individual gross income data, i.e. this variable was not equivalised for household income or composition, as this data were not available for the study. It could have been interesting to see if another definition of income had affected the results.

We also recognise that the model specification applied for indirect standardisation assumes that our variables reflect the concept of need. But just like any study on equity, our results are probably biased by unobservable variation in need correlated with income [52].

## Conclusions

This study, based on a large sample of infection diagnoses, shows that the introduction of digital services in Swedish primary care did not support attainment of equality in health utilization across income. When digital services increase in scope and scale, public governing and purchasing bodies need to find ways to ensure primary care overall is consumed without consideration of the patient’s ability-to-pay. Socio-economic factors are at play, but in different ways depending on type of contact and probably also by medical problem. To formulate effective policy for this, further research is warranted to understand how developing digital services, increasingly integrated with office-based services, and growing digital literacy among patients, affect the utilization pattern. Supply side factors like how office-based and digital services are organised and under what conditions they work should also be included.

## Data Availability

The data used in the current study are available from the corresponding author on reasonable request.

## Footnotes

1 *p*-values < 0.001 for all estimates of concentration indexes.
